# Relationship Between Filament‐Polymerizing Muscle Myosin and Droplets Generated by Liquid–Liquid Phase Separation

**DOI:** 10.1002/cbic.202600006

**Published:** 2026-04-30

**Authors:** Tatsuyuki Waizumi, Mahito Kikumoto, Tomoharu Matsumoto, Kingo Takiguchi

**Affiliations:** ^1^ Department of Biological Science Graduate School of Science Nagoya University Furo‐cho, Chikusa‐ku, Nagoya, Aichi Japan

**Keywords:** droplet deformation, filament polymerization, liquid–liquid phase separation, muscle myosin, protrusion formation of droplet

## Abstract

From individual living cells to the tissues and organs of multicellular organisms, the internal fluids contain numerous macromolecules such as nucleic acids and proteins. Liquid–liquid phase separation (LLPS) is proving to be useful in understanding macromolecular‐mediated phenomena that occur within cells, which have been hard to explain through conventional interactions between biological factors. This LLPS is applicable not only to phenomena at scales equivalent to cells but also to those at much larger scales. We studied the association between the behaviors of myosin, a representative molecular motor isolated from muscle, and LLPS in a binary polymer solution using polyethylene glycol and dextran. Myosin localized in the droplets of the dextran‐rich phase and polymerized to filaments and formed larger assemblies regardless of whether the salt strength did or did not allow polymerization. Those assemblies resembled a coarse mesh of tangled myosin filaments. Conversely, when myosin was inside, the droplets deformed into a non‐spherical morphology. Notably, at a salt strength where myosin normally polymerizes to filaments, some of the deformed droplets even produced sharp protrusions. These findings suggest that not only LLPS modifies the behavior of myosin but also, conversely, myosin affects the nature of the droplets formed by LLPS.

## Graphical abstract

A droplet formed by liquid–liquid phase separation (LLPS) that has deformed and even developed a protrusion due to the inclusion of meshwork structures formed by the assembly of myosin filaments. So far, such droplet deformation has not been observed and was not possible with either DNA or actin filaments. At the same time, myosin was localized inside the droplet and enhanced the ability for polymerization due to the effects of LLPS.

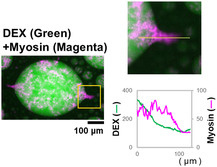

## 1 Introduction

Living cells contain a wide variety of biological factors including high concentrations of macromolecules such as nucleic acids and protein complexes, which can cause liquid–liquid phase separation (LLPS) [1, 2, 3]. LLPS has attracted considerable attention because it has been shown to explain many biological phenomena that were previously hard to understand through conventional perspectives, such as discussions based on the structure and function of individual biological factors or knowledge of unique interactions between specific molecules [4, 5, 6].

By adopting the concepts of phase separation and phase transition, including LLPS, the mechanisms by which various robust systems necessary for life are generated across a wide range of size scales and multiple hierarchical structures through the summing up of molecular self‐organization have come to be understood. Representative examples in which LLPS is thought to play a central role are as follows: the formation and maintenance of membrane‐less organelles that lack physical boundaries such as biological membranes [7, 8]; cellular malfunctions or diseases triggered by protein denaturation or aggregation [9, 10, 11]; sensing physical conditions such as temperature and pressure [12, 13]; and the orchestrated spatial sorting, alignment, or behavior synchronization of populations of numerous biological factors or living cells across mesoscopic to macroscopic scales. Those processes should be the basis by which individual nm‐sized molecules spontaneously construct μm‐sized cellular structures and even larger structures such as tissues, organs, and individuals that range from a millimeter to a meter in size [14, 15, 16, 17, 18]. Thus, the functions of LLPS are diverse. It should be noted that, for these reasons, LLPS is considered one of the essential phenomena involved in the origin of life.

Regarding the last example mentioned above, there have been reports of cases in which the effects of LLPS play an important role in the functions of cytoskeletons or their regulatory and collaborating factors [17, 18]. Muscles are the most prominent example of a system involving the cytoskeleton and its collaborating factors, as well as being a representative biological device that manifests across different scales and hierarchies, from molecules to cells to tissues and organs [19]. Muscles, particularly skeletal muscles, are characterized by the enormous structures formed by the fusion of many cells, their orderly structure consisting of repeated sarcomeres, their synchronized contraction, their robustness against external stimuli, and their ability to self‐repair when damaged. The main components of muscles are actin, the representative cytoskeleton, and myosin, the molecular motor that generates the sliding movement of actin filaments.

The combinations of cytoskeletons and molecular motors that make up the locomotor apparatus of living organisms include sets of microtubules and dynein or kinesin, which are responsible for eukaryotic flagella or cilia and chromosome segregation. However, our research focused on the combination of actin and myosin. Monomeric actin has a size of approximately 5 nm, and in the case of type II myosin (myosin II) used here has a length of 160 nm. Both actin and myosin II can be isolated in large amounts from skeletal muscle, and each of these has the ability to polymerize to form filaments. They coordinate and cooperate “naturally” to assemble structures suited to a wide range of tasks, from the generation of forces required at the individual cellular scale, such as cell movement, cytokinesis, morphological changes, and the traffic of intracellular cargoes, to smooth muscles that support organ function, and skeletal and cardiac muscles, which perform the movements required at the individual organism scale mentioned above [19, 20, 21, 22]. What are those mechanisms? The clustering of individual molecules involved in a reaction, confinement within a finite space the size of a cell, and interaction with lipid membranes have been examined as possible candidates for the mechanism and reveal that these processes are effective in diversifying the structure and function of actin‐ and myosin‐based assemblies [23, 24, 25, 26].

We considered also LLPS to be promising candidate and investigated its effects. As a first step in this approach, our previous studies focused on actin. Those studies revealed that LLPS promotes actin polymerization and the assembly of bundles of actin filaments moved into dextran (DEX)‐rich droplets, even in the presence of a factor that severs actin filaments and obstructs actin polymerization, resulting in morphological changes of the droplets [14, 27]. As part of the same approach, this study focused on another key player, myosin. Myosin II was used in this study and is a protein complex with a molecular weight (MW) of approximately 500 kDa, consisting of two heavy chains and four light chains, and has a radish sprouts‐like shape. Myosin II has long been known to polymerize to form filaments called ‘thick filaments’ in solution conditions close to physiological salt strength, such as 100 mM KCl at a neutral pH [20, 28]. Those myosin filaments can polymerize not only in the longitudinal direction but also in the lateral direction, so they are thicker, with no limit to their thickness, and are stiffer, compared to actin filaments formed by the polymerization of actin, which are sometimes referred to as “thin filaments.” In contrast, at high salt strength conditions, for example 600 mM KCl, myosin II exists alone in a solubilized state and does not polymerize into filaments. Especially within the sarcomeres of skeletal muscles, myosin filaments are accompanied by factors that regulate their size and position or that act as elastic springs or mechanical reinforcements and that cause muscle contraction by generating the sliding of actin filaments [19, 20]. In addition, within the cell, the non‐muscle type II myosin, which has the same overall molecular structure as the skeletal muscle type, although the mechanisms of regulating its activity are different, produces the force and movement required by cells together with various regulatory factors as well as actin filaments [21]. Of course, for energy supply, for the maintenance of system homeostasis, and for repair, a number of other factors cooperate additionally. It is noted here that myosin II has a double‐headed structure with two motor domains called heads. Such double‐headed myosin structures can induce sliding between two actin filaments, resulting in contraction/elongation or bending of bundles of actin filaments, which are formed by the depletion effect in the presence of polymers, as well as sorting actin filaments according to their polarity [23, 29].

An aqueous two‐phase system (ATPS) consisting of soluble polymers with various structures and properties, such as polyethylene glycol (PEG) and DEX, is commonly used to investigate the relevance and effect of LLPS. Similarly, many studies have attempted to utilize ATPS to develop self‐assembly models of cellular structures, including the reconstruction of artificial membrane‐less cell organelles [30, 31, 32, 33, 34, 35]. In this study, in order to investigate the mutual effects between LLPS, which leads to segregation in solution, and myosin II, which has the ability to polymerize filaments as described above, we observed the behavior of myosin in an ATPS, a mixture of PEG and DEX. Here, we will discuss the relationship between LLPS and myosin and their significance for self‐organization mechanisms important for cells or living organisms to develop.

## 2 Results and Discussion

### 2.1 Droplets and Myosin when Myosin Is Added to a PEG/DEX Solution at Salt Strengths that Allow Myosin Polymerization

**FIGURE 1:**
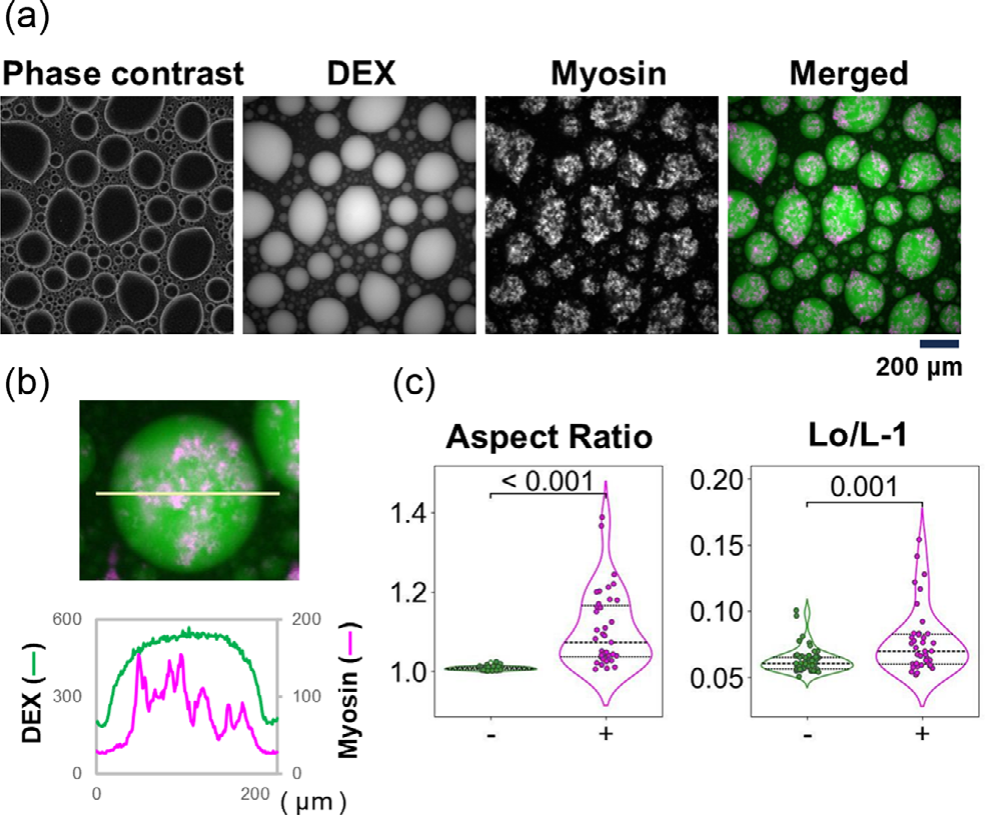
Myosin in the PEG/DEX solution under polymerizable conditions (100 mM KCl). (a) Images of phase contrast, DEX fluorescence, myosin fluorescence, and a merged image of these are shown. These images were obtained 10 min after mixing the solutions. In the merged image, the distribution of DEX and myosin is indicated with green and magenta, respectively. (b) The upper panel shows a fluorescence image of a typical droplet. The lower panel shows its fluorescence intensity profile. The intensity of fluorescence derived from DEX (vertical axis, left) or myosin (right) measured along the position indicated by the yellow line in the image above is shown. (c) The aspect ratio (left) and Lo/L‐1 (right) of the droplets of the DEX‐rich phase in the absence (−) or presence of myosin (+). The value “Lo/L‐1” is a parameter of shape distortion. The same applies to other Figures. The details including the calculation method are described in the Experimental Section. The average, standard deviation, and sample size (Av.  ± S.D., *n*), for each data, from left to right in the order they appeared in Figure, are 1.008 ± 0.005, *n* = 57 and 1.105 ± 0.095, *n* = 39 (for aspect ratio), and 0.063 ± 0.009, *n* = 57 and 0.077 ± 0.025, *n* = 39 (for Lo/L‐1), respectively.

At physiological salt strength, myosin polymerizes to form thick filaments. The solution used in this study with a KCl concentration of 100 mM mimics that condition. Under that condition, almost all of the myosin added to the PEG/DEX solution localized within the droplets of the DEX‐rich phase (Figure 1a). Even though the DEX concentration within the droplets appeared to be constant, the distribution of myosin inside the droplets was uneven, with some areas where myosin appeared to be absent, to areas where many myosin filaments with various sizes were present, and areas where large assemblies of myosin were present (Figure 1b). The assemblies of myosin appeared to be coarse meshworks with various shapes and sizes, suggesting that they were formed by the entanglement of myosin filaments.

In parallel, the droplets with myosin localized inside showed morphological changes from spheres, which is their usual shape. In this study, the morphological changes of the droplets were evaluated using four indices: the aspect ratio for the degree of elongation, the “Lo/L‐1” for the shape distortion, the roundness for the degree of shape unevenness, and the circularity. All of those indices showed that the droplets were clearly deformed (Figures 1c and S1).

**FIGURE 2:**
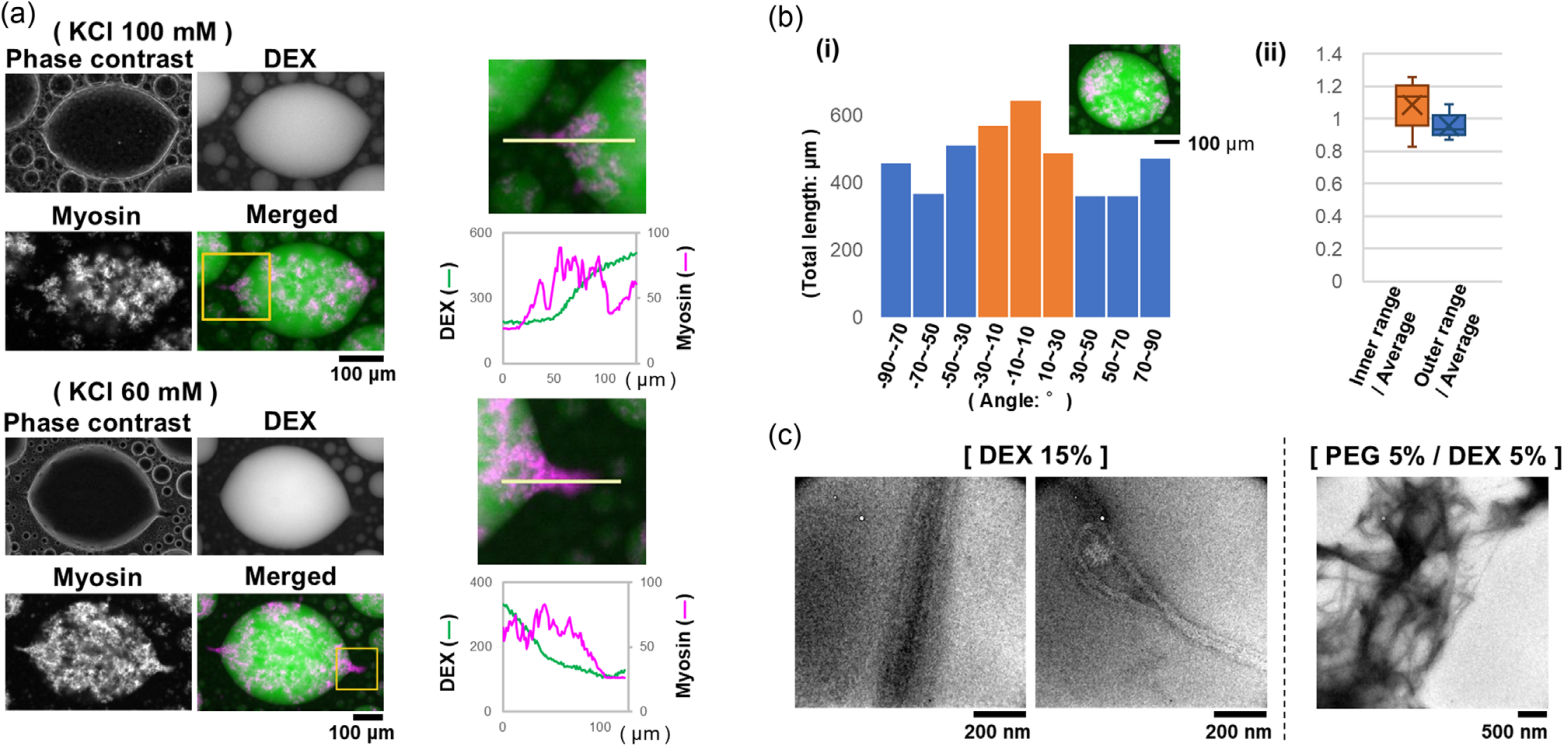
Protrusion formation from deformed droplets. (a) The upper and lower images in the left column are examples of droplets observed in 100 and 60 mM KCl, respectively. Images of phase contrast, DEX fluorescence, myosin fluorescence, and a merged image of those are shown, as in Figure 1a. The right column shows the fluorescence intensity profile of myosin distribution along the position indicated by the yellow line in the area around the protrusion, indicated by the yellow square in the corresponding merged image on the left, as in Figure 1b. (b) Comparison of the long axis direction of the deformed droplet with the alignment direction of the myosin filament assemblies within it. (i) Example of a droplet where the two directions are relatively consistent. (ii) Comparison between the total length (the integral of length and number) of myosin filaments whose orientation is within the inner range (−30° to 30° relative to the long axis of the droplets) and those within the outer range (−90° to −30° and 30° to 90° relative to the long axis of the droplets) shown using the ratio to the average over the entire angle intervals (−90° to 90° relative to the long axis of the droplets). These are the box‐and‐whisker plots of the analysis results for eight droplets. The results for the inner and outer ranges are shown in orange (Av. ± S.D. is 1.085 ± 0.150) and blue (0.957 ± 0.075), respectively. Also see other examples shown in Figure S2. (c) Myosin was added to the 15% DEX or the PEG/DEX solutions and was then observed by electron microscopy. The DEX concentration of the former solution (15%) was decided based on that in the DEX‐phase determined in a previous study [27]. The KCl concentration of the solutions was 100 mM.

In addition, at solution conditions with 60 mM KCl, which allows myosin to polymerize, as in the experiment described above, almost all myosin localized inside the droplets, forming mesh‐work‐like assemblies (Figure 2a). The majority of droplets deformed, presumably due to the presence of myosin assemblies inside, were close to oval sphere‐like shapes.

### 2.2 Protrusion Formation of Deformed Droplets

Some of the droplets that deformed presumably due to the presence of myosin filaments and their assemblies inside resembled spindle‐like shapes rather than oval sphere‐like shapes (Figure 2a). Such droplets even possessed sharp protrusions. Note here that these protrusions were observed regardless of whether the salt strength was 60 or 100 mM KCl, as long as myosin polymerization was possible. The protrusions were often oriented along the long axis of the spindle‐shaped droplet or along the protruding direction of a convex portion of the deformed droplet, such as the vertex of a polygon. Where a protrusion was formed, always myosin, presumably an array of myosin filaments, was observed to be localized perpendicularly to the boundary between the DEX‐rich and PEG‐rich phases, overlapping with the protrusion, and appears to protrude from the droplet of the DEX‐rich phase.

It should be noted that the protrusions of the deformed DEX‐rich droplets found here are unique to myosin. In the case of actin, LLPS promoted polymerization, leading to the formation of actin filaments and their bundles inside the droplets of the DEX‐rich phase, which resulted in droplet deformation, but did not lead to the formation of protrusions [14, 27]. As for DNA, even though long double‐stranded DNA or DNA complexes, e.g., prepared by DNA‐origami, were also incorporated into the droplets, not only did they not form protrusions in the droplets but they also did not deform them [14, 15, 16, 30]. These differences among myosin, actin, and DNA are likely simply due to differences in the thickness and elasticity of each type of filament. If so, other cytoskeletons, microtubule, intermediate filaments, and septin, and other protein/peptide filaments including amyloid fibrils, and even more broadly, various glycans, may also affect with LLPS each other according to their respective properties [14, 16, 18, 19].

Since there appears to be a relationship between the long axis direction of droplets deformed by myosin and the orientation of the protrusions formed, and since myosin is essential for the formation of protrusions, we next investigated the relationship between the morphology of the deformed droplets, such as the orientation of oval sphere‐ or spindle‐like shapes and the alignment of myosin filaments and their assemblies (Figures 2b and S2). The results obtained suggest that there is a subtle, albeit weak, correlation between the orientation of myosin within the droplet and the direction of droplet deformation (two‐sided *t*‐test, *p* = 0.056). Taken together with the finding that myosins were always present where protrusions formed, and with the fact that the solution conditions were at salt strengths that allow for myosin polymerization, it is likely that the droplet deformation observed here is responsible for the thick filaments formed by myosin polymerization. Supporting this prediction, electron microscopy revealed the formation of very long fibers, likely due to further longitudinal assembly of myosin filaments, and a meshwork formed by their entanglement, in a solution mimicking the DEX concentration inside the droplets and in the PEG/DEX solution (Figure 2c).

### 2.3 Droplets and Myosin when Myosin Is Added to the PEG/DEX Solution at a Salt Strength that Does Not Allow Myosin Polymerization

**FIGURE 3:**
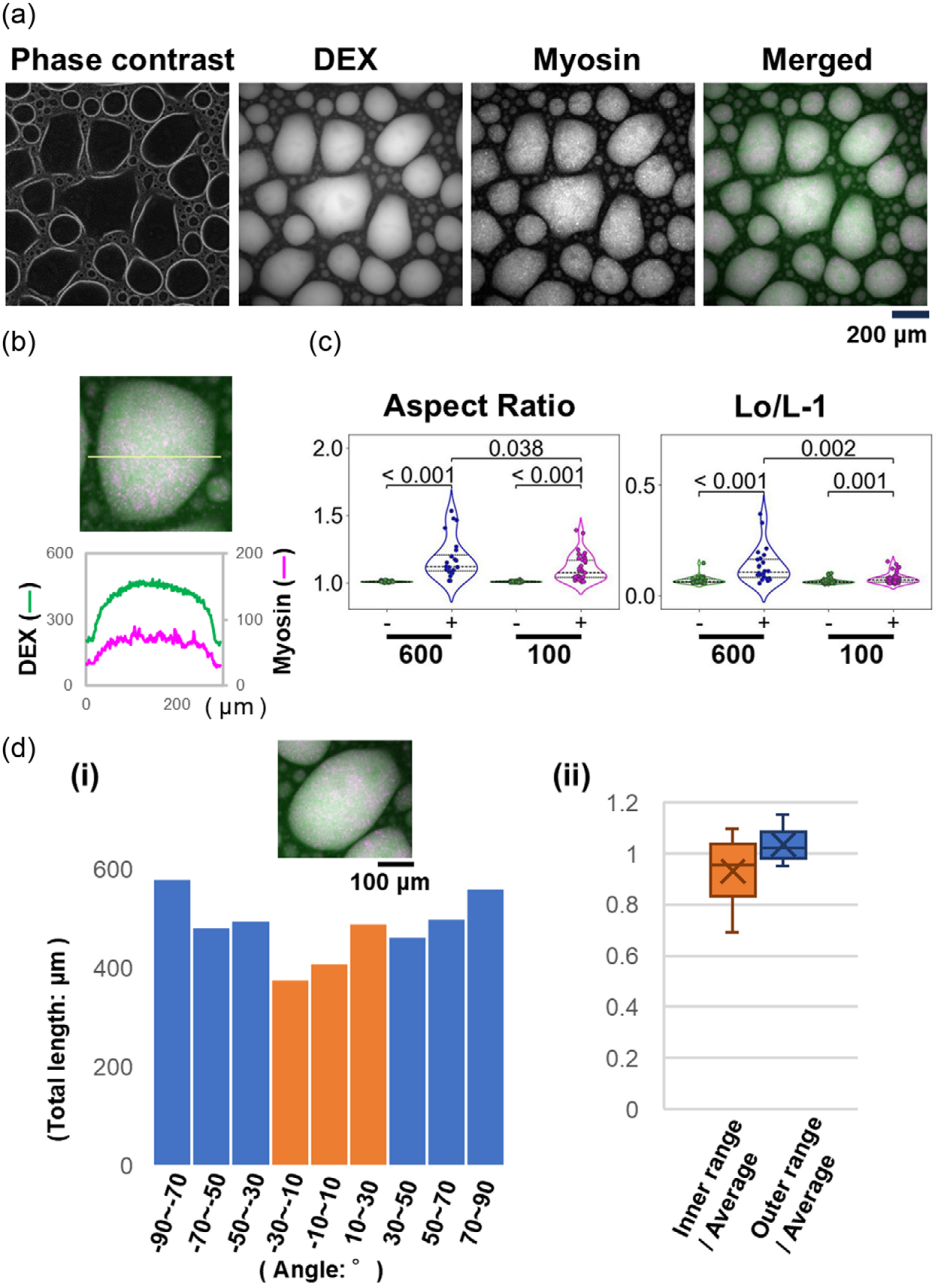
Myosin in a PEG/DEX solution under non‐polymerizable conditions (600 mM KCl). (a) Images of phase contrast, DEX fluorescence, myosin fluorescence, and a merge of those images are shown, as in Figure 1a. (b) The upper panel shows a fluorescence image of a typical droplet. The lower panel shows the fluorescence intensity profile derived from DEX (vertical axis, left) or myosin (right) measured along the position indicated by the yellow line in the image above, as in Figure 1b. (c) The aspect ratios (left) and Lo/L‐1 (right) of the droplets in the absence (−) or presence of myosin (+) are shown. For comparison, the results obtained in the condition of 100 mM KCl (shown in Figure 1c, please note that the scales of the vertical axes are different) are shown again. The average, standard deviation, and sample size for each data of 600 mM, from left to right in the order they appeared in Figure, are 1.008 ± 0.005, *n* = 34 and 1.178 ± 0.148, *n* = 24 (for aspect ratio), and 0.068 ± 0.016, *n* = 34 and 0.134 ± 0.079, *n* = 24 (for Lo/L‐1), respectively. (d) Comparison of the long axis direction of the deformed droplet with the alignment direction of the myosin filament assemblies within it. (i) An example of results for a deformed droplet. (ii) Comparison between the total length (the integral of length and number) of myosin filaments whose orientation is within the inner range and those within the outer range shown using the ratio to the average over the entire angle intervals, as in Figure 2b. These are the box‐and‐whisker plots of the analysis results for six droplets. The results for the inner and outer ranges are shown in orange (Av. ± S.D. is 0.933 ± 0.140) and blue (1.034 ± 0.070), respectively (two‐sided *t*‐test, *p* = 0.157).

For comparison, the same experiments as described above were conducted at a salt strength where myosin would normally not polymerize. For that condition, a solution with a KCl concentration of 600 mM was used. Even under the high salt strength, myosin in the PEG/DEX solution localized within the droplets of the DEX‐rich phase and unexpectedly formed filaments (Figure 3a and b). At this high salt strength, however, myosin was relatively uniformly localized inside the droplets. This is thought to be because the individual myosin filaments were smaller, and therefore, the assemblies formed by their entanglements were also smaller, resulting in finer mesh‐works, which is consistent with this condition which would normally not allow myosin to polymerize.

Even under this condition, especially over time, droplet deformations presumably due to the accumulation into inside and subsequently the formation of filaments or assemblies of myosin were observed. In comparison to the results obtained at conditions with 100 mM or less KCl, myosin induces more diverse morphological changes other than a spindle‐like shape to the droplets under this high salt strength. Indeed, the parameters of droplet morphology obtained in the condition of 600 mM KCl show that not only were droplets significantly deformed from a spherical shape but also that the shapes of the deformed droplets were different from the case with myosin added at 100 mM KCl (Figures 3c and S3). Furthermore, as an important morphological difference, the droplet protrusions observed at low salt strengths were not observed at this high salt strength.

We compared the long axis direction of the deformed droplets or the direction in which they were most elongated, with the orientation of the alignments of myosin filaments and assemblies inside, also at 600 mM KCl. At this high salt strength, no correlation was observed (Figure 3d). Therefore, the mechanism or process behind the droplet deformation caused by the addition of myosin appears to differ between 100 and 600 mM KCl concentrations. It is thought that myosin, which existed in an individually solubilized state because the concentration of KCl was 600 mM, may gradually polymerize to filaments and subsequently form assemblies independently in various locations inside a droplet, due to the accumulation and polymerization induced by LLPS. Probably, the myosin filaments or assemblies formed in this way are not only small, but are also localized inside the droplets as lacking anisotropy and without any geometrical feature, leading to a relatively uniform distribution. As a result, the droplets are thought to exhibit a variety of irregular deformations that were polygon‐like and differed from the spindle shape.

**FIGURE 4:**
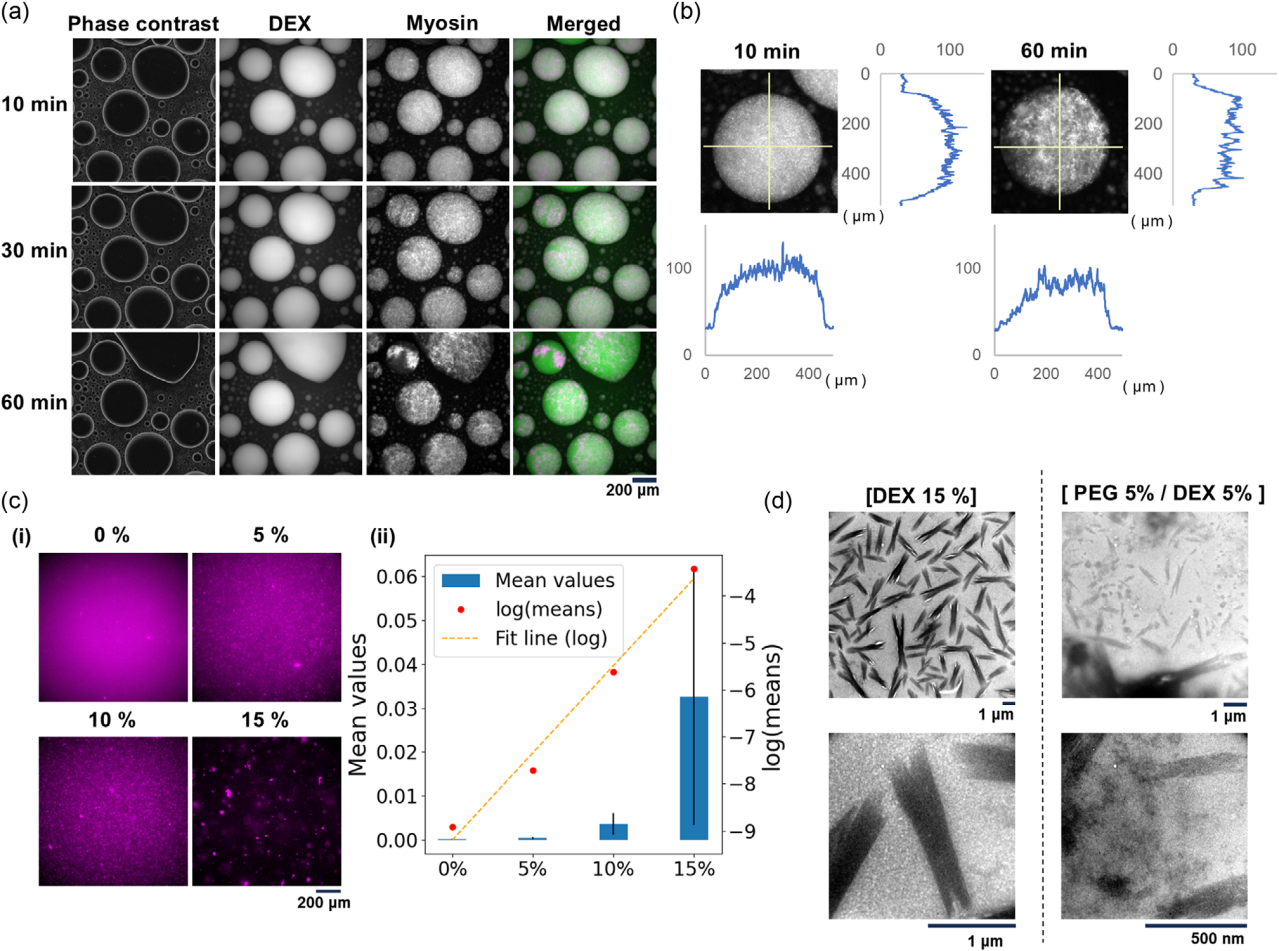
Myosin in the presence of DEX under non‐polymerizable conditions (600 mM KCl). (a) Time course images observed when solubilized myosin was added to the PEG/DEX solution while maintaining the final KCl concentration at 600 mM. Images of phase contrast, DEX fluorescence, myosin fluorescence, and a merged image of these are shown 10, 30, and 60 min after mixing. (b) Intensity profiles of myosin fluorescence for a typical droplet after 10 and 60 min. In both cases after 10 and 60 min, the upper left panel shows a fluorescence image of a typical droplet. The right and lower panels show the fluorescence intensity profiles measured along the position indicated by the yellow lines in the corresponding images. (c) Myosin in the presence of different concentrations of DEX. (i) Examples of myosin fluorescence images. The DEX concentration is indicated above each image. (ii) The amount of myosin forming filaments and assemblies. For each fluorescence image, the fluorescence intensity and number of pixels in the areas which show fluorescence intensity above a threshold (set uniformly across all images) were integrated, and this was then divided by the product of the average fluorescence intensity and the total number of pixels (total area). This panel shows the mean (left vertical axis, bar graph, error bars show S.D.) and the logarithm of the mean (right vertical axis, line graph) value calculated as above for each DEX concentration (below each graph or point). For each data of the bar graph, from 0 to 15% of DEX concentrations, 0.000135 ± 0.000175 (*n* = 4), 0.000448 ± 0.000240 (*n* = 4), 0.00363 ± 0.00242 (*n* = 6), and 0.0326 ± 0.0291 (*n* = 5), respectively. (d) Myosin was added to the 15% DEX or the PEG/DEX solutions and was then observed by electron microscopy. The KCl concentration of the solutions was 600 mM.

To confirm this prediction, while maintaining the final KCl concentration at 600 mM, the time course of myosin in the PEG/DEX solution was observed from after its addition (Figure 4a). Notably, almost immediately after mixing, most of the myosin was located in the DEX‐rich phase, i.e., the inside of the droplets. Meanwhile, as expected, myosin polymerization inside the droplets proceeded gradually. Initially, the distribution of myosin appeared nearly uniform throughout the droplet, but over time, filaments or their assemblies were observed (Figure 4b).

The promoted polymerization at solution conditions where polymerization would not normally occur has also been observed in the case of actin [14, 27]. It is thought that due to the depletion effect that works in a crowded environment of polymers, the equilibrium between polymerization and depolymerization was significantly shifted toward polymerization, and the accumulation in the DEX‐rich phase was induced at the same time. Here, to investigate the case of myosin, we observed the state of myosin in DEX solutions at the condition of 600 mM KCl (Figure 4c). Myosin polymerization increased monotonically with increasing DEX concentration, and all myosin was polymerized at 15% DEX, which is roughly equivalent to the DEX concentration in the droplets of the DEX‐rich phase as determined in previous studies [27]. These results indicate that, similar to the case of actin, myosin is concentrated in the DEX‐rich phase due to the effect of LLPS, and polymerization is promoted by DEX, resulting in the formation of filaments, even under conditions of high salt concentration where polymerization would normally not occur.

In addition, electron microscopy revealed that the structures of myosin filaments formed by the effects of LLPS even at a high salt strength were short and thick, apparently formed by side‐to‐side adhesions of multiple myosin filaments (Figure 4d).

### 2.4 Myosin Filaments and the Droplet Deformations

The formation and spherical shape of droplets in the PEG/DEX solution did not differ at different salt strengths, at least when no protein was added (Figure S4). Therefore, the differences in droplet deformations and protrusion formations observed at different salt strengths are due to differences in the state of the myosin filaments inside the droplets. Here, the mechanism of droplet deformations will be discussed, focusing on the characteristics of filaments formed by proteins such as myosin, and comparing them with actin filaments.

In the case of actin, a large number of actin filaments was located along the boundary with the outside or at the periphery, rather than the interior parts of the droplets. The actin filaments along the boundary may reduce the curvature of the boundary, resulting in a polyhedral‐like shape of the droplets, or may prevent the completion of fusion between the droplets that come into contact, making these beaded [14, 27]. On the other hand, myosin, other than being involved in protrusion formation, predominantly formed meshworks in the comparably inner parts of the droplet. These features of myosin distribution are markedly different from the case of actin filaments (Figures 1–4).

Inside the droplets of the DEX‐rich phase, not only at 100 mM KCl but also at 600 mM KCl, myosin polymerized to form filaments, and assemblies and/or meshwork structures of these filaments formed, resulting in the droplet deformations (Figures 1 and 3). Under low salt strengths such as 100 mM KCl, which allow myosin polymerization, longer and comparably larger‐sized myosin filaments were formed (Figure 2). Since the meshwork made of such myosin filaments grows larger (as well as coarser), some may appear that can affect the entire droplet. In such cases, by feedback between the mechanical structure of the meshwork and the overall shape of the droplet, the deformation of the droplet and the reorientation of the myosin filaments that make up the meshwork may occur in parallel with a bias in the same direction. As a result, the droplets would tend to deform to oval sphere‐like shapes. Furthermore, myosin filaments themselves and their bundles are thicker and should be much stiffer than actin filament bundles, so they would be able to overcome the tension acting at the boundary of the droplets that actin filament bundles could not overcome, making it possible for them to extend in the perpendicular direction that leads to the protrusion formation. Under high salt strength such as 600 mM KCl, stocky and comparably smaller‐sized myosin filaments were formed (Figure 4). The meshworks made of such myosin filaments are fine‐meshed, but small in size, and can only affect a limited part of the droplet. Still, as mentioned above, these myosin filaments and their assemblies may be able to overcome the tension acting on the droplet boundary, causing the part of the droplet to deform in its own direction, independently of the deformation in other parts. As a result, the droplets are thought to exhibit a variety of irregular deformations such as polygon‐like or distorted shapes.

As discussed above, we have considered the relationship between myosin filament assembly and droplet morphogenesis based on a comparison of the structure of myosin filaments and the bundles or assemblies they form. However, direct measurements of the size, shape, and stiffness of myosin filaments, as well as the elasticity, interfacial tension, and viscoelastic response of droplets, have not yet been performed. If these mechanical properties can be measured and quantitatively analyzed, it will help identify the key physical parameters that govern droplet morphogenesis. Furthermore, if theoretical analysis can be performed and combined with experimental research, we can deepen our understanding of the mechanism by clarifying the relationship between the characteristics of myosin, or of its fragments (those described below), and the droplet deformation manner (*e.g.*, determining whether it is elliptical or polygonal, or whether protrusion is formed) at the molecular or mesoscale level. Indeed, attempts to perform molecular dynamics simulations or construct coarse‐grained models of peptide behavior under LLPS have been actively pursued in recent years, so that these approaches will be expanded to systems consisting of actin and myosin [36, 37].

### 2.5 Region of Myosin Required for Droplet Deformations

**FIGURE 5:**
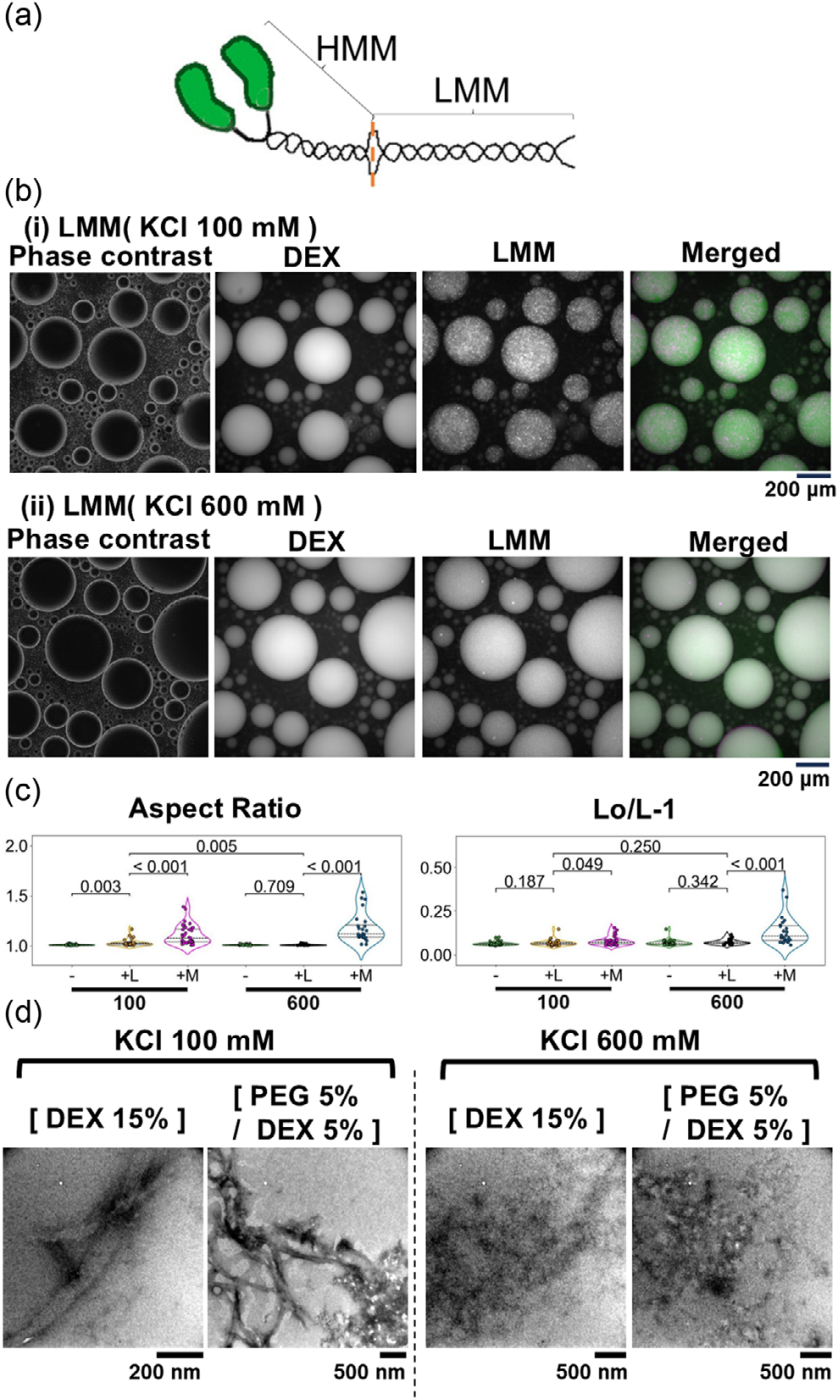
Results when LMM was added instead of myosin. (a) Model of the molecular structure of myosin (Myosin II) showing the 2 proteolytic fragments used in this study, the LMM and HMM regions. Two heavy chains are connected in a coiled‐coil structure. The green areas indicate the heads of the motor domain. Please note that for simplicity, this model only shows the part of heavy chains and omits the four light chains. (b) LMM in the PEG/DEX solution. (i) and (ii) are images of phase contrast, DEX fluorescence, LMM fluorescence, and a merged image of those observed in 100 and 600 mM KCl, respectively. (c) The aspect ratio (left) and Lo/L‐1 (right) of the droplets in the absence (−) or presence of LMM (indicated by “+ L”) at 100 and 600 mM KCl are shown. For comparison, in addition to the control results without any added protein, the results obtained in the presence of myosin (shown in Figures 1 and 3, please note that the scales of the vertical axes are different) are shown again (indicated by “+ M”). The average, standard deviation, and sample size for each data obtained in the presence of LMM are 1.027 ± 0.034, *n* = 33 (100 mM) and 1.008 ± 0.007, *n* = 25 (600 mM) for aspect ratio, and 0.067 ± 0.018, *n* = 33 (100 mM) and 0.072 ± 0.015, *n* = 25 (600 mM) for Lo/L‐1, respectively. The roundness and circularity of droplets containing LMM are shown in Figure S5. (d) LMM was added to the 15% DEX or the PEG/DEX solutions, and was then observed by electron microscopy. The KCl concentrations of the solutions were 100 or 600 mM, as shown in each image.

To investigate whether the formation of filaments by the polymerization of myosin is solely responsible for the behavior observed in myosin and the droplets, experiments were also carried out using two types of fragments obtained by the proteolytic cleavage of myosin. One fragment is called light meromyosin (LMM) and consists of the region necessary and sufficient for myosin polymerization and filament formation, while the other fragment is called heavy meromyosin (HMM) and contains the motor domains called the heads for sliding actin filaments and the region called the neck that continues from the heads (Figure 5a).

When LMM, instead of myosin, was added to the PEG/DEX solution, it localized in the droplets of the DEX‐rich phase regardless of the salt strength, and clear filamentous structures formed inside the droplets especially at the low salt strength of 100 mM KCl (Figure 5b). However, the droplets remained nearly spherical (Figures 5c and S5). The results that were significantly different from the control were only the aspect ratio and roundness obtained at 100 mM KCl, in consistent with the condition under which filamentous structures were observed (Figure 5b and d). Because LMM is a fragment of myosin, its molecule is short (Figure 5a), and it is possible that this is the reason why droplet deformation did not occur. So that, to compensate, a higher concentration of LMM was added, but the results remained unchanged (Figure S6). In the case of myosin, significant differences were observed in all of the parameters, regardless of the KCl concentration, as mentioned above. Therefore, the difference in droplet deformation ability between myosin and LMM suggests that factors other than protein filament formation may be involved in the droplet deformation caused by myosin.

**FIGURE 6:**
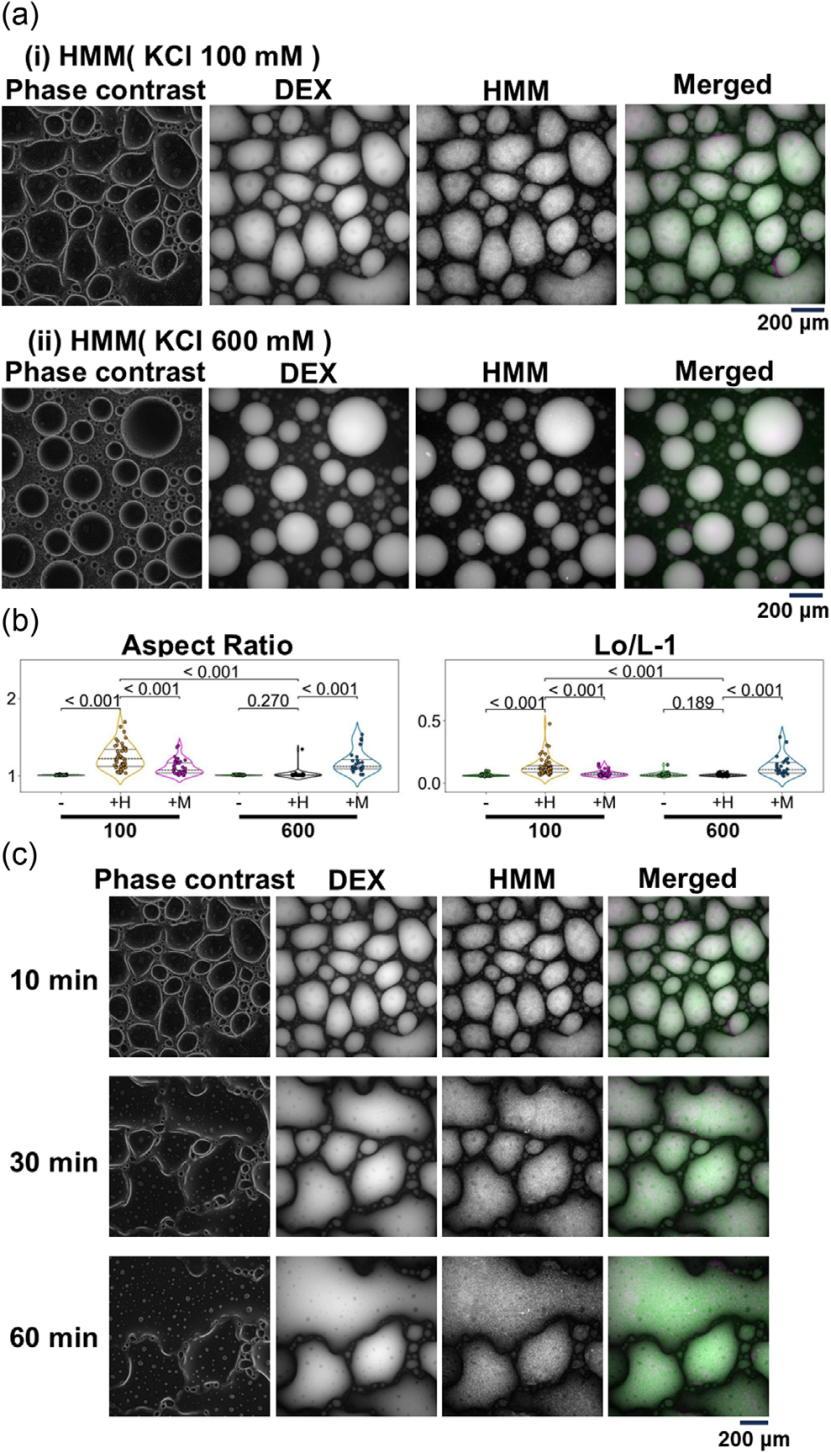
Results when HMM was added instead of myosin. (a) HMM in the PEG/DEX solution. (i) and (ii) are images of phase contrast, DEX fluorescence, HMM fluorescence, and a merged image of those in 100 and 600 mM KCl, respectively. (b) The aspect ratio (left) and Lo/L‐1 (right) of the droplets in the absence (−) or presence of HMM (indicated by “+ H”) at 100 and at 600 mM KCl are shown. For comparison, in addition to the control results without any added protein, the results obtained in the presence of myosin (shown in Figures 1 and 3, please note that the scales of the vertical axes are different) are shown again (indicated by “+ M”). The average, standard deviation, and sample size for each data obtained in the presence of HMM are 1.242 ± 0.159, *n* = 50 (100 mM) and 1.017 ± 0.054, *n* = 38 (600 mM) for aspect ratio, and 0.133 ± 0.075, *n* = 50 (100 mM) and 0.064 ±  0.009, *n* = 38 (600 mM) for Lo/L‐1, respectively. The roundness and circularity of droplets containing HMM are shown in Figure S7. (c) Time course images observed when HMM was added to the PEG/DEX solution at 100 mM KCl. Images are shown for each time period up to 60 min as indicated. Images shown are the same as in (a).

HMM affected the droplets only at the salt strength of 100 mM KCl, but not at 600 mM KCl (Figure 6a). When 100 mM KCl was used, all parameters of droplet morphology revealed significant differences against both the control and the case when myosin was added (Figures 6b and S7). On the other hand, at 600 mM KCl, HMM uniformly localized inside the droplets of the DEX‐rich phase, but did not affect the shape or behavior of the droplets. Indeed, for all parameters of droplet morphology, there was no significant difference from the control. The results of observations over time at a salt strength of 100 mM KCl showed that HMM that concentrated inside the DEX‐rich droplets tended to cause droplet coalescence and/or to obscure the boundary between the PEG‐rich and DEX‐rich phases, rather than being interpreted as deformation of the droplets (Figure 6c). Since the effect of HMM clearly depended on the salt strength of the solution, it was possible that it resulted from an electrostatic effect derived from the large number of basic amino acid residues in HMM. To verify this, poly‐L‐lysine (PLL), a peptide composed solely of the basic residue lysine, was used instead of HMM, and the behavior of the droplets was observed. However, PLL did not show any effect (Figure S8). Thus, the effect of HMM on the LLPS does not appear to simply result from the charge of the protein fragment.

Both proteolytic fragments of myosin failed to show the same effect as intact myosin, indicating that the entire myosin molecule is important for the relationship between myosin and the effects of LLPS observed in this study. Note here that it has been reported that skeletal muscle myosin, when a mutation was made to remove the heads, can form filaments in vitro but fails to properly assemble into the sarcomere structure of muscle in vivo [20]. This may have some relevance to our findings.

It should be noted here that a report showing that droplets of the DEX‐rich phase accumulate cations [38]. This effect would direct the DEX‐rich droplets to tend to adhere to negatively charged glass surface, which could lead to their deformation. If this is the case, then increasing the salt concentration of the solution should induce droplet deformation due to enhance the effect. However, the results obtained here show that, the droplets kept their spherical shape without depending on the salt strength (Figure S4), and additionally in the presence of PLL (Figure S8), at least in our experimental conditions. The deformations of the droplets were observed only when whole myosin molecules were added (Figures 1–3), and the protrusion formation, which might be the most noticeable deformation, occurred only rather at low salt concentrations (Figure 2), indicating that myosin is responsible for the observed droplet deformations.

## 3 Conclusion

From living cells to the tissues and organs of multicellular organisms, the internal fluids contain numerous macromolecules such as nucleic acids and proteins. LLPS is proving to be useful in understanding macromolecular‐mediated phenomena that occur at scales equivalent to or greater than the cell size, which have been hard to explain through conventional interactions between biological factors. We studied the association between the behaviors of myosin, a representative molecular motor isolated from muscle, and LLPS in a binary polymer solution using PEG and DEX.

The findings in this study are as follows: Myosin concentrated inside the droplets of the DEX‐rich phase, regardless of whether the salt strength does or does not allow polymerization. Not only at 100 mM KCl but also at 600 mM KCl, myosin polymerized to form filaments, and assemblies and/or meshworks of these filaments inside the droplets. Longer and larger filaments were observed at 100 mM KCl, while short and stocky filaments were observed at 600 mM KCl probably due to side‐to‐side adhesions of multiple myosin filaments. Conversely, as myosin assemblies were inside, the droplets deformed to non‐spherical shapes, i.e., oval spheres at 100 mM KCl and a variety of irregular deformations such as polygon‐like shapes at 600 mM KCl. Notably, only at 100 mM KCl where myosin normally polymerizes to filaments, some of the deformed droplets even possessed sharp protrusions. The features of the structure of myosin filaments polymerized inside the droplets seem to be consistent with the morphology of the deformed droplets. Indeed, the orientation of myosin filaments and their assemblies inside the deformed droplets showed, although subtle, a relationship with the long axis direction of the deformed droplet only at 100 mM KCl. In addition, the deformations of droplets caused by myosin required the entire molecule. Thus, the phenomenon found in this study is not simply a system that depends on the formation of filaments/assemblies of proteins, but must be underpinned by a complex mechanism that also requires some other effect(s).

These results suggest that LLPS not only modifies the behavior of myosin, the molecular motor working in muscles, but also conversely, myosin would affect the nature of the cell‐sized or much larger scale droplets that are formed in polymer solutions under crowded condition and depletion effects. While these results are still far from clarifying the mechanisms by which molecules automatically assemble into large complex tissues such as muscle fibers or regular periodic structures such as sarcomeres, we believe that this research has contributed an important first step toward understanding them. As a next step, it should be essential to investigate the functions co‐generated by actin filaments and myosins and the phenomena that arise from their coexistence, not only simply in the presence of polymers, but also in an environment where the effects of LLPS and compartmentalization of the solution by droplets are at work [23, 29]. An additional crucial issue to address is the generality of the observed myosin‐induced droplet deformations and protrusion formation, that is, whether the principles are directly applicable to actual biological environments. In this study, we used an ATPS consisting of PEG and DEX with a MW combination frequently used in LLPS research [30, 31, 32, 33, 34, 35]. By expanding the scope of analysis, such as by changing the MWs of PEG and DEX, or by using alternative polymers or LLPS systems, thereby the biological significance of the knowledge obtained in this study about how the crowding and depletive effects of polymers correlate with myosin polymerization and droplet morphogenesis would be enhanced.

Lastly, in regard to the mutually unique phases generated in a solution by LLPS, the deformation of a droplet, especially the production of protrusions discovered in this study, could lead to connecting separate regions of the same phase through thin channels, or regulating the shape and area of the boundaries between different two phases. Concerning the thin channel, examples of structures of “connecting passages” that play important roles in living organisms and that are thought to involve the cytoskeleton/molecular motors and/or LLPS, include dendritic spines in neurons, plasmodesmata in plant cells, and nuclear pores, among many others [39, 40, 41, 42, 43].

## 4 Experimental Section

### 4.1 Reagents and Solutions

We used an ATPS consisting of PEG and DEX as previously described [14, 15, 27]. PEG 6,000 was purchased from FUJIFILM Wako Pure Chemical Industries (Osaka, Japan); its average MW was 7,300 to 9,300 Da. DEX was also purchased from FUJIFILM Wako Pure Chemical Industries; its average MW was 180,000 to 210,000 Da. For use as a tracer for DEX‐rich domains, fluorescein isothiocyanate‐labeled DEX (FITC‐DEX) was purchased from Sigma (St. Louis, MO, USA). For use as a tracer for PEG‐rich domains, methoxyl PEG fluorescein (mPEG‐Fluorescein) was purchased from Nanocs Inc. (New York, NY, USA). These polymers were dissolved in Milli‐Q water (18.2 MΩ cm) to prepare stock solutions (10–30 wt%). For the preparation of each sample, a mixture of PEG and DEX was agitated just before mixing with the other solutions. Other reagents of special grade or higher for biochemistry were purchased from FUJIFILM Wako Pure Chemical Industries, and stock solutions were prepared using Milli‐Q water.

The final conditions of the sample solutions were 5% PEG, 5% DEX, 5 mM Tris‐HCl (pH 8.0), 0.2 mM DTT, 2 mM MgCl_2_, 2 mM ATP, 0.9 mM NaHCO_3_, 60, 100, or 600 mM KCl, and 0.5 μM myosin or its fragment, LMM or HMM. When confirming phase separation using a fluorescence image, 4.9% DEX or PEG was mixed with 0.1% FITC‐DEX or mPEG‐Fluorescein, respectively. Salt strength, that is, the concentration of KCl in each experiment, or protein concentration in experiments with proteins at a concentration different than that mentioned above is indicated in the Figure captions.

### 4.2 Proteins

A stock solution of 50% glycerinated myosin was prepared from rabbit skeletal muscle as described previously [44]. HMM and LMM were digested from glycerinated myosin using the preparation method for HMM with chymotrypsin [45]. For LMM, it was further separated by polymerization‐depolymerization cycles [46, 47]. Myosin and its fragments were fluorescently labeled with Cy‐5 dye (COSMO BIO, Tokyo, Japan) under solubilizing conditions, specifically a high ionic strength condition (600 mM KCl) for myosin and LMM. Unmodified dyes were removed by two polymerization/depolymerization cycles followed by dialysis for myosin and LMM, and by dialysis for HMM. Note here that this dye did not affect the LLPS and the droplet formation, especially the spherical morphology of the droplets of the DEX‐rich phase, and it even tended to distribute in the PEG‐rich phase (Figure S9). This indicates that protein samples fluorescently labeled with this reagent can be used without worrying about side effects. In all experiments, myosin and its fragments were dialyzed against and kept in a solution with a KCl concentration of 600 mM, and then mixed with other solutions.

As above, the proteins required for this study were isolated and purified from rabbits. We hereby declare that ethical approval for animal experiments was obtained from Nagoya University (Graduate School of Science, Nagoya University; approval numbers: S220005‐001, S230001‐002, S240001‐001, and S250001‐001), and the study was conducted according “The Guidelines for Proper Conduct of Animal Experiments” established by the Science Council of Japan.

### 4.3 Optical Microscopy

Images were obtained using phase contrast and fluorescence microscopes (BX60/IX70, Olympus, Tokyo, Japan/BX53, Evident, Tokyo, Japan) with a 40× objective (NA = 0.75, Olympus) attached to a polarizing unit and a camera (WAT‐910HX, Watec, Tsuruoka, Japan/IR‐1000, DAGE‐MTI, Michigan City, IN, USA/ORCA Flash 4.0V3, Hamamatsu Photonics, Hamamatsu, Japan). The acquired images were recorded in HDD using a PC [27]. The program ImageJ (http://imagej.nih.gov/ij/) was used to adjust the contrast and to analyze images as described below.

The solutions of the mixture of PEG/DEX and myosin or its fragment were placed into each microchamber made from a slide glass and a double‐sided seal (SLF0601 Frame‐Seal 15 × 15 mm 65 μL, BIO‐RAD (Hercules, CA, USA), and then sealed with a coverslip, prior to microscopic observations.

### 4.4 Electron Microscopy

A 2 μL drop of each sample was applied to a glow‐discharged carbon‐coated copper grid. After waiting 1 min to allow settling, excess solution was blotted, and the specimen was stained with 2% uranyl acetate. Specimens were observed using a H‐7600 transmission electron microscope (Hitachi High‐Tech Co., Tokyo, Japan) operated at 100 kV acceleration. Images were recorded with an Orius SC200D CCD camera (Gatan, Inc., Pleasanton, CA, USA).

### 4.5 Image Analysis

Morphological changes of droplets were evaluated using the following parameters: the aspect ratio (the length ratio of the long axis and the short axis), the “Lo/L‐1” (the perimeter actually measured from its cross‐sectional image divided by the theoretical perimeter calculated from the area actually measured from the same image, and then subtracted by 1), the roundness (4 times the area actually measured from its cross‐sectional image divided by the square of its long axis length and π), and the circularity (4π times the area actually measured from its cross‐sectional image divided by the square of the perimeter actually measured from the same image). Each data are presented as a violin plot with lines indicating the median and interquartile range.

To evaluate droplet morphology as described above, the DEX‐derived fluorescence images were binarized and measured using ImageJ (Figure S10). It should be noted that if the size of the droplet of the DEX‐rich phase is small, *i.e.*, the fluorescent image equivalent to its cross‐sectional image is small, the perimeter measured using ImageJ becomes larger than the actual length, likely due to a discrepancy between the resolution and the pixel size of the image, making accurate evaluation difficult. In order to address this issue while minimizing artifacts, we set a threshold of area according to the following procedure and analyzed only droplets larger than that size. First, under control experimental conditions, i.e., in the absence of myosin, droplets only appeared circular, while from these images, the perimeter measured without any processing and the perimeter measured after approximating the shape to an ellipse were obtained, and the ratio of the former to the latter was plotted against the area of each droplet. The plot resembled a decay curve that asymptotically approached 1. This curve was considered to be the sum of the results from two groups: small droplets that would have a perimeter longer than the actual length, and droplets large enough to analyze using ImageJ. Here, for convenience, the plot was subtracted by 1 and then fitted to the sum of two exponential curves, and the area where the two curves intersect was defined as the threshold (Figure S11).

The angle of orientation of filaments and assemblies of myosin within a droplet was obtained by extracting their edges using ImageJ, binarizing the image, removing overlapping areas between different filaments or assemblies, and then skeletonizing the image to fit an ellipse to determine the angle. The angle determined by the above procedure was compared with the long axis direction of the droplet (graphs were drawn in nine groups of 20° each, centered on the long axis direction of the droplet). The angle of the long axis direction of the droplet was determined from its image that was binarized and approximated to an ellipse using ImageJ.

### 4.6 Statistical Analysis

All results reported are based on at least three independent experimental runs. Statistical analyses were conducted with SciPy, including Welch's *t*‐test. The probability (P) values are indicated to show statistical significance in Figures, and the average and standard deviation (Av. ± S.D.), and sample size (n) of each data are described in the Figure captions. Figures were generated using matplotlib/Excel, and final layouts were adjusted using PowerPoint/GIMP.

## Author Contributions

T.W. and K.T. conceived this project. T.W. conducted the experiments and analyzed and compiled the results. M.K. prepared the protein‐related materials and confirmed their activity, and T.M. conducted the electron microscopy observations. K.T. wrote the manuscript with feedback from the coauthors.

## Supporting Information

Additional supporting information can be found online in the Supporting Information section.

## Funding

This study was supported by the MEXT KAKENHI Japan (JP2520H05972), JSPS KAKENHI Japan (JP19K06540) and THERS Make New Standards Program for the Next Generation Researchers (JST SPRING) Japan (JPMJSP2125 (Nagoya Univ., Japan)).

## Conflicts of Interest

The authors declare no conflicts of interest.

## Supporting information

Supplementary Material

## Data Availability

The data that support the findings of this study are available from the corresponding author upon reasonable request.

## References

[1] S. Asakura and F. Oosawa , “On Interaction between Two Bodies Immersed in a Solution of Macromolecules,” The Journal of Chemical Physics 22 (1954): 1255–1256.

[2] S. Asakura and F. Oosawa , “Interaction between Particles Suspended in Solutions of Macromolecules,” Journal of Polymer Science 33 (1958): 183–192.

[3] C.‐Y. Shew , S. Oda , and K. Yoshikawa , “Localization Switching of a Large Object in a Crowded Cavity: A Rigid/Soft Object Prefers Surface/Inner Positioning,” The Journal of Chemical Physics 147 (2017): 204901. 29195278 10.1063/1.5000762

[4] C. P. Brangwynne , P. Tompa , and R. Pappu , ““Polymer Physics of Intracellular Phase Transitions,” Nature Physics 11 (2015): 899–904.

[5] D. M. Mitrea and R. W. Kriwacki , “Phase Separation in Biology; Functional Organization of a Higher Order,” Cell Communication and Signaling 14 (2016): 1. 26727894 10.1186/s12964-015-0125-7 PMC4700675

[6] S. Alberti , A. Gladfelter , and T. Mittag , “Considerations and Challenges in Studying Liquid‐Liquid Phase Separation and Biomolecular Condensates,” Cell 176 (2019): 419–434. 30682370 10.1016/j.cell.2018.12.035 PMC6445271

[7] E. M. Courchaine , A. Lu , and K. M. Neugebauer , “Droplet Organelles?,” The Embo Journal 35 (2016): 1603–1612. 27357569 10.15252/embj.201593517 PMC4969579

[8] V. N. Uversky , “Intrinsically Disordered Proteins in Overcrowded Milieu: Membrane‐Less Organelles, Phase Separation, and Intrinsic Disorder,” Current Opinion in Structural Biology 44 (2017): 18–30. 27838525 10.1016/j.sbi.2016.10.015

[9] A. von Appen , D. La Joie , I. E. Johnson , et al., “LEM2 Phase Separation Promotes ESCRT‐Mediated Nuclear Envelope Reformation,” Nature 582 (2020): 115–118. 32494070 10.1038/s41586-020-2232-x PMC7321842

[10] N. M. Kanaan , C. Hamel , T. Grabinski , and B. Combs , “Liquid‐Liquid Phase Separation Induces Pathogenic Tau Conformations in Vitro,” Nature Communications 11 (2020): 2809. 10.1038/s41467-020-16580-3 PMC7272632 32499559

[11] Y. Shin and C. P. Brangwynne , “Liquid Phase Condensation in Cell Physiology and Disease,” Science 357 (2017): eaaf4382. 28935776 10.1126/science.aaf4382

[12] J.‐H. Jung , A. D. Barbosa , S. Hutin , et al., “A Prion‐Like Domain in ELF3 Functions as a Thermosensor in *Arabidopsis* ,” Nature 585 (2020): 256–260. 32848244 10.1038/s41586-020-2644-7

[13] K. Watanabe , K. Morishita , X. Zhou , et al., “Cells Recognize Osmotic Stress through Liquid–liquid Phase Separation Lubricated with Poly(ADP‐Ribose),” Nature Communications 12 (2021): 1353. 10.1038/s41467-021-21614-5 PMC7921423 33649309

[14] N. Nakatani , H. Sakuta , M. Hayashi , et al., “Specific Spatial Localization of Actin and DNA in a Water/Water Microdroplet: Self‐Emergence of a Cell‐Like Structure,” ChemBioChem 19 (2018): 1370–1374. 29676062 10.1002/cbic.201800066 PMC6055874

[15] H. Sakuta , F. Fujita , T. Hamada , et al., “Self‐Emergent Protocells Generated in an Aqueous Solution with Binary Macromolecules Through Liquid‐Liquid Phase Separation,” ChemBioChem 21 (2020): 3323–3328. 32667694 10.1002/cbic.202000344 PMC7754443

[16] K. Tsumoto , H. Sakuta , K. Takiguchi , and K. Yoshikawa , “Nonspecific Characteristics of Macromolecules Create Specific Effects in Living Cells,” Biophysical Reviews 12 (2020): 425–434. 32144739 10.1007/s12551-020-00673-w PMC7242541

[17] K. Tsukita , M. Kitamata , H. Kashihara , et al., “Phase Separation of an Actin Nucleator by Junctional Microtubules Regulates Epithelial Function,” Science Advances 9 (2023): eadf6358. 36791197 10.1126/sciadv.adf6358 PMC9931218

[18] T. Higa , S. T. Kijima , T. Sasaki , et al., “Microtubule‐Associated Phase Separation of MIDD1 Tunes Cell Wall Spacing in Xylem Vessels in *Arabidopsis Thaliana* ,” Nature Plants 10 (2024): 100–117. 38172572 10.1038/s41477-023-01593-9

[19] B. Alberts , A. Johnson , J. Lewis , et al., Molecular Biology of the Cell, 6th ed. (W. W. Norton and Company, 2015), 889–962.

[20] K. Ojima , “Myosin: Formation and Maintenance of Thick Filaments,” Animal Science Journal 90 (2019): 801–807. 31134719 10.1111/asj.13226 PMC6618170

[21] D. E. Rassier and A. Månsson , “Mechanisms of Myosin II Force Generation: Insights from Novel Experimental Techniques and Approaches,” Physiological Reviews 105 (2025): 1–93. 38451233 10.1152/physrev.00014.2023

[22] M. A. Hartman and J. A. Spudich , “The Myosin Superfamily at a Glance,” Journal of Cell Science 125 (2012): 1627–1632. 22566666 10.1242/jcs.094300 PMC3346823

[23] K. Takiguchi , “Heavy Meromyosin Induces Sliding Movements between Antiparallel Actin Filaments,” Journal of Biochemistry 109 (1991): 520–527. 1869506 10.1093/oxfordjournals.jbchem.a123414

[24] R. Sakamoto , M. Tanabe , T. Hiraiwa , et al., “Tug‐of‐War between Actomyosin‐Driven Antagonistic Forces Determines the Positioning Symmetry in Cell‐Sized Confinement,” Nature Communications 11 (2020): 3063. 10.1038/s41467-020-16677-9 PMC7295813 32541780

[25] Y. Sato , R. Sumiyoshi , M. Yamagishi , et al., ”Reconstructing the Motility Driven by Membrane‐Bound Myosin on the Inner Surface of Cell‐Sized Droplets,” Langmuir 41 (2025): 10077, 40238146 10.1021/acs.langmuir.4c04123

[26] Y. Nishigami , H. Ito , S. Sonobe , and M. Ichikawa , “Non‐Periodic Oscillatory Deformation of an Actomyosin Microdroplet Encapsulated Within a Lipid Interface,” Scientific Reports 6 (2016): 18964. 26754862 10.1038/srep18964 PMC4709586

[27] T. Waizumi , H. Sakuta , M. Hayashi , K. Tsumoto , K. Takiguchi , and K. Yoshikawa , “Polymerization/Depolymerization of Actin Cooperates with the Morphology and Stability of Cell‐Sized Droplets Generated in a Polymer Solution Under a Depletion Effect,” The Journal of Chemical Physics 155 (2021): 075101. 34418942 10.1063/5.0055460

[28] S. Ebashi and F. Ebashi , “A New Protein Component Participating in the Superprecipitation of Myosin B,” Journal of Biochemistry 55 (1964): 604–613. 14216404 10.1093/oxfordjournals.jbchem.a127933

[29] Y. Tanaka‐Takiguchi , T. Kakei , A. Tanimura , et al., “The Elongation and Contraction of Actin Bundles Are Induced by Double‐Headed Myosins in a Motor Concentration‐Dependent Manner,” Journal of Molecular Biology 341 (2004): 467–476. 15276837 10.1016/j.jmb.2004.06.019

[30] M. Masukawa , Y. Sato , F. Yu , K. Tsumoto , K. Yoshikawa , and M. Takinoue , “Water‐in‐Water Droplets Selectively Uptake Self‐Assembled DNA Nano/Microstructures: A Versatile Method for Purification in DNA Nanotechnology,” ChemBioChem 23 (2022): e202200240. 35686962 10.1002/cbic.202200240 PMC9544409

[31] Y. Chao and H. C. Shum , “Emerging Aqueous Two‐Phase Systems: From Fundamentals of Interfaces to Biomedical Applications,” Chemical Society Reviews 49 (2020): 114–142. 31750468 10.1039/c9cs00466a

[32] K. Tsumoto and K. Yoshikawa , “The Aqueous Two Phase System (ATPS) Deserves Plausible Real‐World Modeling for the Structure and Function of Living Cells,” MRS Advances 2 (2017): 2407–2413.

[33] C. D. K. W. M. Aumiller Jr. , “Experimental Models for Dynamic Compartmentalization of Biomolecules in Liquid Organelles: Reversible Formation and Partitioning in Aqueous Biphasic Systems,” Advances in Colloid and Interface Science 239 (2017): 75–87. 27401136 10.1016/j.cis.2016.06.011

[34] J. Esquena , “Water‐in‐Water (W/W) Emulsions,” Current Opinion in Colloid & Interface Science 25 (2016): 109–119.

[35] M. Iqbal , Y. Tao , S. Xie , et al., “Aqueous Two‐Phase System (ATPS): An Overview and Advances in Its Applications,” Biological Procedures Online 18 (2016): 18. 27807400 10.1186/s12575-016-0048-8 PMC5084470

[36] N. Galvanetto , M. T. Ivanović , A. Chowdhury , et al., “Extreme Dynamics in a Biomolecular Condensate,” Nature 619 (2023): 876–883. 37468629 10.1038/s41586-023-06329-5 PMC11508043

[37] S. Shi , L. Zhao , and Z.‐Y. Lu , “Coarse‐Grained Modeling of Liquid–Liquid Phase Separation in Cells: Challenges and Opportunities,” The Journal of Physical Chemistry Letters 15 (2024): 7280–7287. 38979955 10.1021/acs.jpclett.4c01261

[38] H. Sakuta , Y. Akamine , A. Kamo , et al., “Cation Accumulation Drives the Preferential Partitioning of DNA in an Aqueous Two‐Phase System,” ACS Macro Letters 15 (2026): 302–308. 41528809 10.1021/acsmacrolett.5c00810 PMC12919576

[39] N. Honkura , M. Matsuzaki , J. Noguchi , G. C. Ellis‐Davies , and H. Kasai , “The Subspine Organization of Actin Fibers Regulates the Structure and Plasticity of Dendritic Spines,” Neuron 57 (2008): 719–729. 18341992 10.1016/j.neuron.2008.01.013

[40] M. D. Rubio , R. Johnson , C. A. Miller , R. L. Huganir , and G. Rumbaugh , “Regulation of Synapse Structure and Function by Distinct Myosin II Motors,” The Journal of Neuroscience 31 (2011): 1448–1460. 21273429 10.1523/JNEUROSCI.3294-10.2011 PMC3074980

[41] A. J. Maule , “Plasmodesmata: Structure, Function and Biogenesis,” Current Opinion in Plant Biology 11 (2008): 680–686. 18824402 10.1016/j.pbi.2008.08.002

[42] M. S. Mohamed , M. Hazawa , A. Kobayashi , et al., “Spatiotemporally Tracking of Nano‐Biofilaments Inside the Nuclear Pore Complex Core,” Biomaterials 256 (2020): 120198. 32622019 10.1016/j.biomaterials.2020.120198

[43] S. Takamori , H. Mimura , T. Osaki , et al., “Nuclear Assembly in Giant Unilamellar Vesicles Encapsulating Xenopus Egg Extract,” Small 21 (2025): e2412126. 40390663 10.1002/smll.202412126 PMC12199120

[44] A. G. Weeds and R. S. Taylor , “Separation of Subfragment‐1 Isoenzymes from Rabbit Skeletal Muscle Myosin,” Nature 257 (1975): 54–56. 125854 10.1038/257054a0

[45] A. G. Weeds and B. Pope , “Studies on the Chymotryptic Digestion of Myosin. Effects of Divalent Cations on Proteolytic Susceptibility,” Journal of Molecular Biology 111 (1977): 129–157. 323500 10.1016/s0022-2836(77)80119-8

[46] A. G. Szent‐Györgyi , C. Cohen , and D. E. Philpott , “Light Meromyosin Fraction I: A Helical Molecule from Myosin,” Journal of Molecular Biology 2 (1960): 133−142, IN1–IN4.

[47] Y. Okamoto and T. Sekine ,“A Streamlined Method of Subfragment One Preparation from Myosin,“ Journal of Biochemistry 98 (1985): 1143–1145. 4077843 10.1093/oxfordjournals.jbchem.a135365

