# Supplementary material for: Relationship Between Filament‐Polymerizing Muscle Myosin and Droplets Generated by Liquid–Liquid Phase Separation
Source: Chembiochem. 2026 Apr 30;27(9):e202600006. doi: 10.1002/cbic.202600006 (PMC13133544; doi:10.1002/cbic.202600006)
Supplement: Supplementary file 1 — Supplementary Material [file CBIC-27-e202600006-s001.pdf]

Supporting Information

Relationship Between Filament–Polymerizing Muscle Myosin and Droplets Generated by Liquid–Liquid Phase Separation

Tatsuyuki Waizumi, Mahito Kikumoto, Tomoharu Matsumoto, and Kingo Takiguchi*[a]

[a] T. Waizumi, Dr. M. Kikumoto, Dr. T. Matsumoto, Dr. K. Takiguchi
Department of Biological Science, Graduate School of Science
Nagoya University
Furo-cho, Chikusa-ku, Nagoya, Aichi 464-8602 (Japan)

Supplementary figures

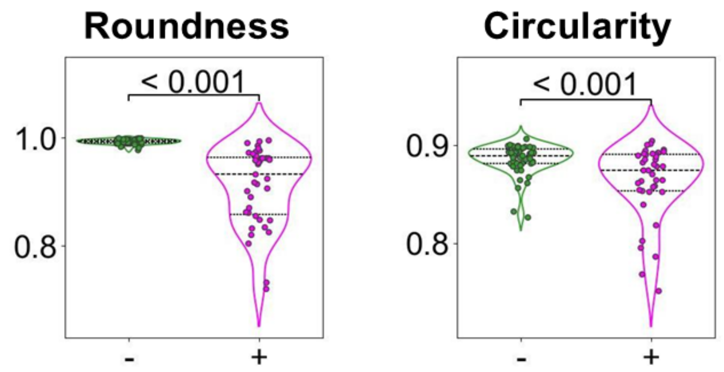

Figure S1. The roundness and circularity of droplets when myosin was added to the PEG/DEX solution under polymerizable conditions (100 mM KCl). The (Av. ± S.D., n) for each data, from left to right in the order they appeared in Figure, are 0.992 ± 0.005, n = 57 and 0.911 ± 0.072, n = 39 (for roundness), and 0.886 ± 0.015, n = 57 and 0.863 ± 0.038, n = 39 (for circularity), respectively.

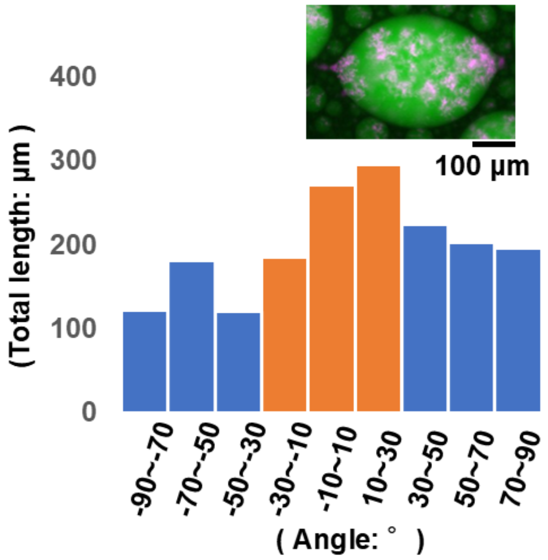

Figure S2. Comparison of the long axis direction of a deformed droplet with the alignment direction of the myosin filament assemblies within it. This is also an example of a droplet where the two directions are relatively consistent.

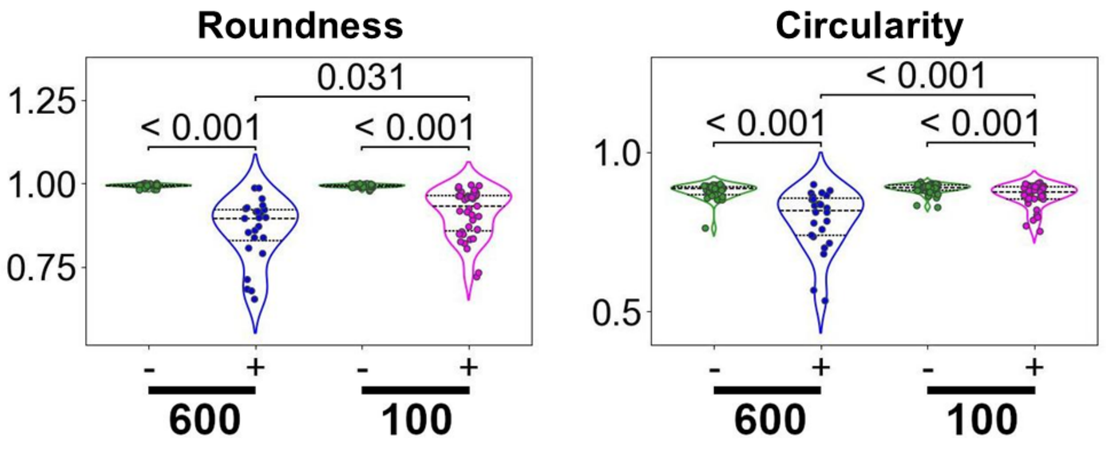

Figure S3. The roundness and circularity of droplets when myosin was added to the PEG/DEX solution under non-polymerizable conditions (600 mM KCl). The (Av. ± S.D., n) for each data of 600 mM are 0.993 ± 0.005, n = 34 and 0.860 ± 0.095, n = 24 (for roundness), and 0.877 ± 0.025, n = 34 and 0.787 ± 0.095, n = 24 (for circularity), respectively.

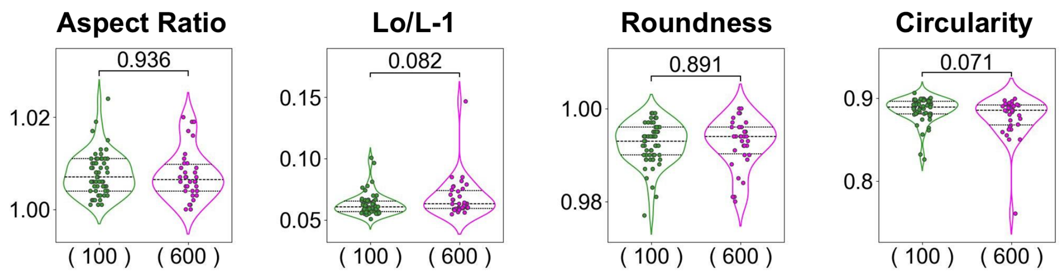

Figure S4. Droplet shape at 100 mM KCl and 600 mM KCl in the absence of myosin or its fragments. In the cases of droplets observed in the control experiment, all four parameters for droplet shape analyzed in this study, aspect ratio, Lo/L-1, roundness, and circularity, were indistinguishable between the two salt strength conditions. The results shown in Figures 1 and 3 and Supplementary Figures S1 and S3 are shown again (please note that the scales of the vertical axes are different). Therefore, the (Av. ± S.D., n) for each data, from left to right in the order they appeared in Figure, are 1.008 ± 0.005, n = 57 (100 mM) and 1.008 ± 0.005, n = 34 (600 mM) for aspect ratio, 0.063 ± 0.009, n = 57 (100 mM) and 0.068 ± 0.016, n = 34 (600 mM) for Lo/L-1, 0.992 ± 0.005, n = 57 (100 mM) and 0.993 ± 0.005, n = 34 (600 mM) for roundness, 0.886 ± 0.015, n = 57 (100 mM) and 0.877 ± 0.025, n = 34 (600 mM) for circularity, respectively.

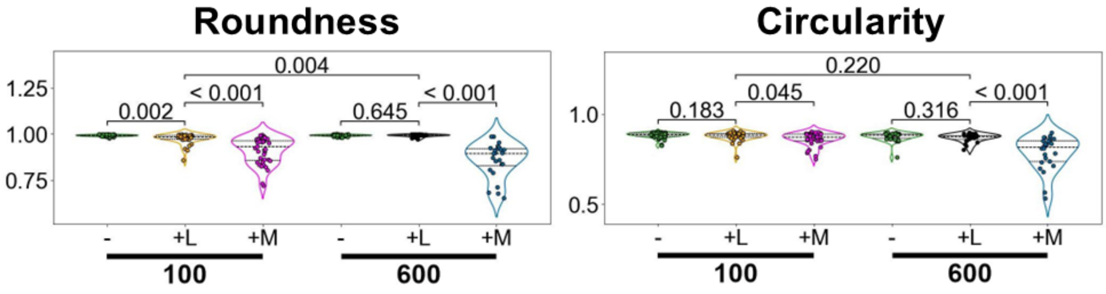

Figure S5. The roundness and circularity of droplets in the absence (-) or presence of LMM (indicated by "+ L") at 100 mM KCl and at 600 mM KCl are shown. For comparison, in addition to the control results without any added protein, the results obtained in the presence of myosin (shown in Figures S1 and S3, please note that the scales of the vertical axes are different) are shown again (indicated by "+ M"). The (Av. ± S.D., n) for each data obtained in the presence of LMM are 0.975 ± 0.030, n = 33 (100 mM) and 0.992 ± 0.007, n = 25 (600 mM) for roundness, and 0.879 ± 0.027, n = 33 (100 mM) and 0.870 ± 0.024, n = 25 (600 mM) for circularity, respectively.

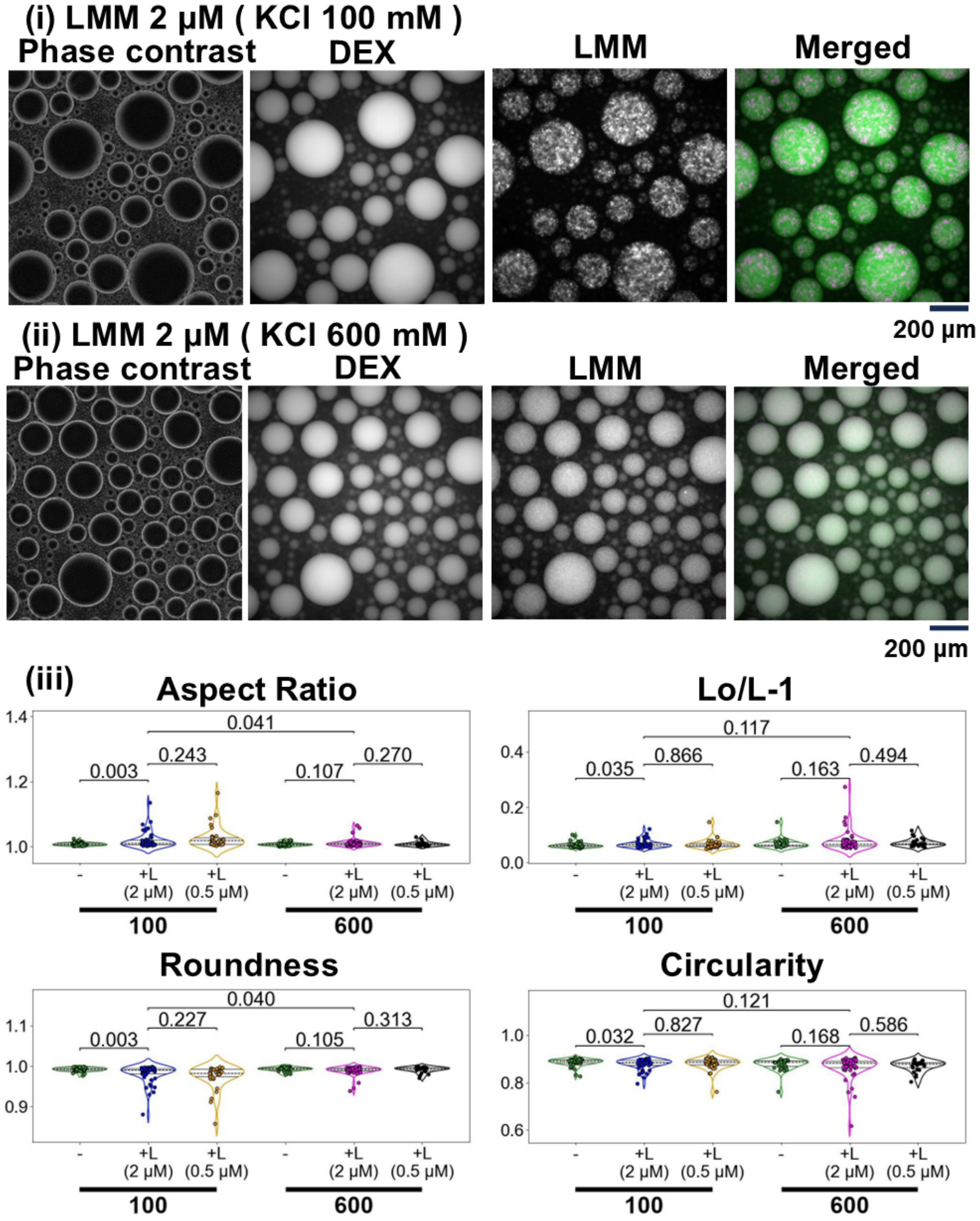

Figure S6. Droplets when a larger amount of LMM was added to the PEG/DEX solution. Microscopic images of droplets and results of analyses for droplet deformation obtained when LMM was added at 2.0 μM are shown, as in Figure 5 (also see Figure S5). The (Av. ± S.D., n) for each data obtained in the presence of 2.0 μM LMM are 1.018 ± 0.024, n = 51 (100 mM) and 1.011 ± 0.012, n = 54 (600 mM) for aspect ratio, 0.068 ± 0.014, n = 51 (100 mM) and 0.076 ± 0.035, n = 54 (600 mM) for Lo/L-1, 0.982 ± 0.022, n = 51 (100 mM) and 0.990 ± 0.011, n = 54 (600 mM) for roundness, 0.878 ± 0.023, n = 51 (100 mM) and 0.866 ± 0.048, n = 54 (600 mM) for circularity, respectively.

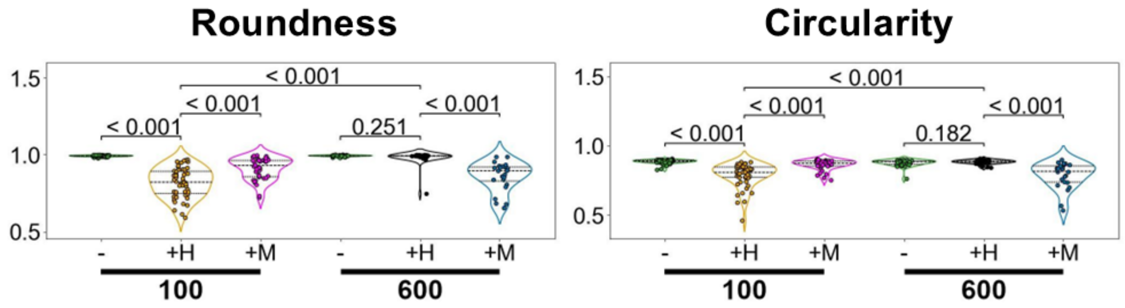

Figure S7. The roundness and circularity of droplets in the absence (-) or presence of HMM (indicated by "+ H") at 100 mM KCl and at 600 mM KCl are shown. For comparison, in addition to the control results without any added protein, the results obtained in the presence of myosin (indicated by "+ M") are shown again (Figures S1 and S3, please note that the scales of the vertical axes are different). The (Av. ± S.D., n) for each data obtained in the presence of HMM are 0.817 ± 0.097, n = 50 (100 mM) and 0.985 ± 0.040, n = 38 (600 mM) for roundness, and 0.788 ± 0.086, n = 50 (100 mM) and 0.884 ± 0.015, n = 38 (600 mM) for circularity, respectively.

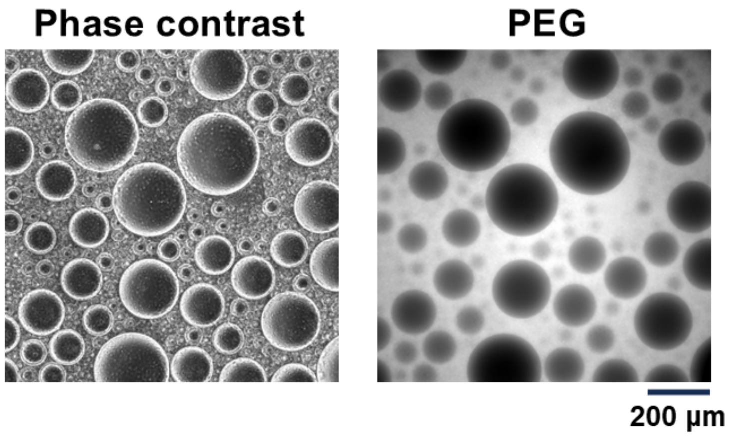

Figure S8. Images of droplets when 0.1 mg/mL PLL, as a convenient multivalent cation, is added to the PEG/DEX solution. Images of phase contrast and PEG fluorescence are shown. The concentration of KCl is 100 mM.

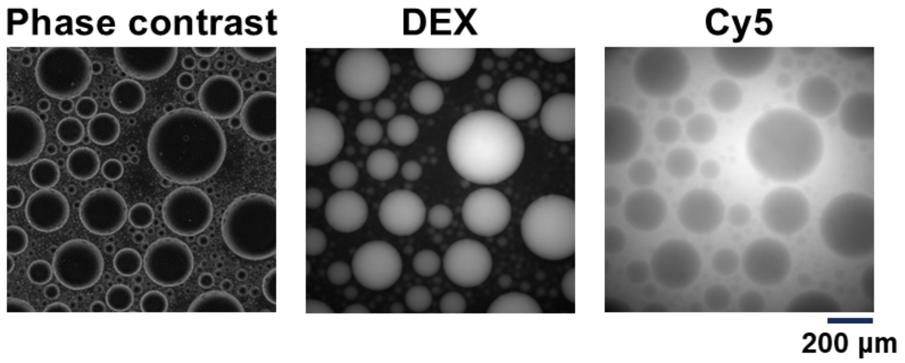

Figure S9. Images of droplets when the fluorescent labeling reagent Cy-5 alone was added to the PEG/DEX solution. Images of phase contrast, DEX fluorescence, and Cy-5 fluorescence, are shown.

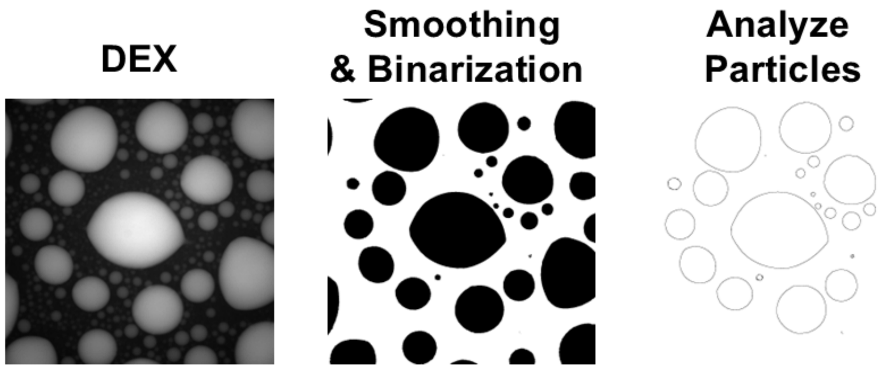

Figure S10. Image analysis procedure. An original fluorescence microscope image (left), its binarized image (center), and the shape of the droplets extracted (right). Then, based on this, measurements and approximations are performed. Noted that the result shown in Figure 2 (a) (KCl 60 mM) is used as an example.

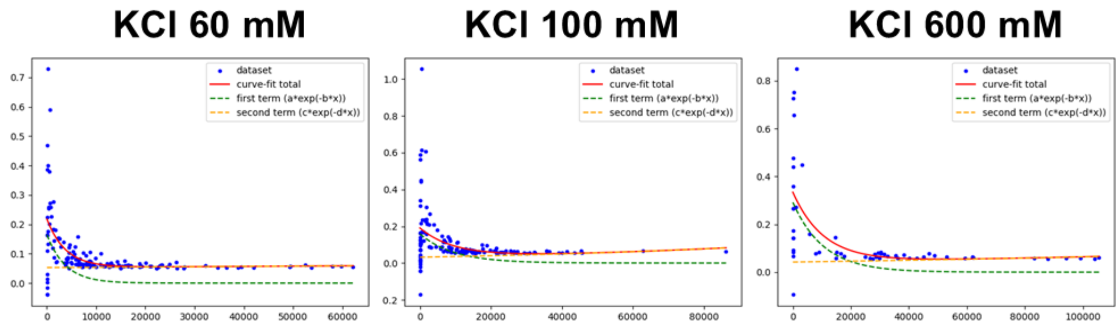

Figure S11. Plots for setting a threshold of droplet size for perimeter length measurement and analysis. From the fluorescence images of the control droplets, the perimeter measured without any processing and the perimeter measured after approximating the shape to an ellipse were obtained, and the ratio of the former to the latter was subtracted by 1, and then plotted against the area of each droplet (at each salt strength indicated). The plot curve (red solid line in each graph) was fitted to the sum of two exponential curves (two dotted lines with different colors), and the area where the two curves intersect was calculated. The average (12,600 μm^2) of the area obtained at each salt strength (60, 100, and 600 mM KCl) was defined as the threshold, since similar areas were obtained even at different salt strengths. Sample sizes (n) were 139 (60 mM), 146 (100 mM), and 55 (600 mM), respectively.
